# Supplementary material for: Research on the emissions from industrial products exported from Guangdong Province—an input-output model analysis
Source: PLoS One. 2022 Nov 3;17(11):e0276300. doi: 10.1371/journal.pone.0276300 (PMC9632880; doi:10.1371/journal.pone.0276300)
Supplement: S1 File — (DOCX) [file pone.0276300.s001.docx]

Highlights

1) An input-output model is used analyze the embodied emissions in Guangdong’s industrial exports.

2)Embodied emissions of the top five industries account for about 80% of total emissions.

3) Wastewater emissions’ technical effect has more impact than scale and structural effects’ impacts while waste gas and solid waste are not.

4) The trends and factors influencing various industries’ pollution emissions differ.
