# Supplementary material for: Research on the emissions from industrial products exported from Guangdong Province—an input-output model analysis
Source: PLoS One. 2022 Nov 3;17(11):e0276300. doi: 10.1371/journal.pone.0276300 (PMC9632880; doi:10.1371/journal.pone.0276300)
Supplement: Attachment — Submitted filename: Response to Reviewers.docx [file pone.0276300.s003.docx]

Response to reviewers’ comments

--------------------------

Please use the space provided to explain your answers to the questions above. You may also include additional comments for the author, including concerns about dual publication, research ethics, or publication ethics. (Please upload your review as an attachment if it exceeds 20,000 characters)

--------------------------

Reviewer #1: The author has highlighted only about waste water emissions what about other pollutants from the industries.

I doubt that adjustment in industrial structure would reduce the emission.

Why the author has not insisted about EMS certification as a control measure

**Response:**

This study uses an input-output model to analyze the wastewater, waste gas, and solid waste emissions found in Guangdong’s industrial exports from 2004 to 2015.We studied not only waste water emissions, but also waste gas, and solid waste emissions.

1.This paper carries out the structural decomposition of the emissions from industrial products exported from Guangdong Province. We suggest that improvement of technical level will reduce the emission significant.

2.On the page14, we add EMS as a control measure to reduce emissions for international trade.

-----------------------------------------------------------------------------------------------------

Reviewer #2: This study investigated the wastewater, waste gas, and solid waste emissions in Guangdong’s industrial exports from 2004 to 2015. The authors use LMDI index and input-output model to analyze the factors. This study classified Guangdong’s foreign trade industries and analyze and compare waste gas emissions, wastewater emissions, solid waste emissions for each category. Overall, the authors have spent a lot of efforts on this study and the manuscript has been prepared in a professional manner. It is well organized and the message is clear. However, there are areas for improvement in terms of content. The current manuscript has a major revision of some issues that need to be addressed before being considered for publication.

1. The unit in figure 1 is not defined,

The manufactured good amount and total export is seemed equal in figure however the proportion of manufactured goods have fluctuation, it’s better that chat shows the differences.

2. The second section (research area and method) lacks a detailed review of the model and methodology

What is the other method and index to analyze the problem?

What is the other methods and index that used in existing literature?

Why authors use input-output model and LMDI to analyze the problem?

3. Most of research in literature review is focused on research conducted in china, it’s proposed to mention and investigate other existing research in other countries.

4. Some parts of the paper can be shorten (e.g., Sections 4) and proposed to summarize some analysis and result in table for readers.

**Response:**

1.We add the unit for figure 1. As the proportion of exported industrial products is greater than 97%. Actually, the amount of manufactured goods is really close to the amount of total export. we just can adjust the Figure 1 to make it more clear.

2.We add detailed review of the model and methodology in the second section which can be seen in P4-P6.

Most papers use input-output approach to analyze this problem. SRIO(Single regional Input-output)\BTIO(Bilateral trade input-output)\MRIO(Multilateral regional input-output) are widely used, this paper just studied the problem of Guangdong Province, so we just used SRIO to analysis the emission of industrial products. SDA(Structural Decomposition Analysis) and LMDI(The logarithmic mean Division index) are widely used to describe the decomposition of pollution emission structure, but LMDI can overcome the cross-term problem of SDA well, so we chose LMDI as the index. Based on your comments, we have clarified and explained the difference of the current study with the available literature, which can be seen in P4-P6

3.We also list some existing research in other countries like OECD countries\American\ Norway\ South Korea\ Poland\ Turkish\ Saudi Arabia, and so on, which can be seen in P4-P5

4. Based on your comments, Sections 4 has been shorten and we summarized analysis and result in table, which can be seen in P11 and table2-3.

----------------------------------------------------------------------------------------------------

Reviewer #3: 1. Keywords should ideally be phrases of 2-4 words. Research keywords are not carefully selected. It is also necessary to give the reader an adequate explanation after choosing a keyword. and change the keywords "LMDI" to "The logarithmic mean Division index".

2. The reason for doing research (research gap) and research innovation must be clearly stated in the abstract and the introduction.

3. It is better to compare the results of this study with other similar studies. Also, the benefits of research are described.

**Response:**

1. Based on your comments, we have selected research keywords which can be seen P2.

2. We have clarified and explained reason for doing research which can be seen in P3. Research innovation has been stated in the introduction which can be seen P3-P4.

3. Due to the different regions studied, we made some comparisons in terms of research methods, which can be seen in p6 Based on your comments, the benefits of research are described in P4.
