# Supplementary material for: Research on the emissions from industrial products exported from Guangdong Province—an input-output model analysis
Source: PLoS One. 2022 Nov 3;17(11):e0276300. doi: 10.1371/journal.pone.0276300 (PMC9632880; doi:10.1371/journal.pone.0276300)
Supplement: Attachment — Submitted filename: Response to Reviewers.docx [file pone.0276300.s004.docx]

Journal Requirements:

Please review your reference list to ensure that it is complete and correct. If you have cited papers that have been retracted, please include the rationale for doing so in the manuscript text, or remove these references and replace them with relevant current references. Any changes to the reference list should be mentioned in the rebuttal letter that accompanies your revised manuscript. If you need to cite a retracted article, indicate the article’s retracted status in the References list and also include a citation and full reference for the retraction notice.

**Response:**

We reviewed our reference carefully, and we haven’t find any papers have been retracted.

-----------------------------------------------------------------------------------------------------

Dear Authors,
The paper is improved but sincerely the conclusions are not clear. I suggest to enhance the entire-section providing managerial implications, main issues and what are the key-messages of your work.

**Response:**

We read our paper carefully and improved it as much as we could. As for the deeper causes of the described problems, we may need to conduct further research and publish in the next paper.

-----------------------------------------------------------------------------------------------------

While revising your submission, please upload your figure files to the Preflight Analysis and Conversion Engine (PACE) digital diagnostic tool, <https://pacev2.apexcovantage.com/>. PACE helps ensure that figures meet PLOS requirements. To use PACE, you must first register as a user. Registration is free. Then, login and navigate to the UPLOAD tab, where you will find detailed instructions on how to use the tool. If you encounter any issues or have any questions when using PACE, please email PLOS at [figures@plos.org](mailto:figures@plos.org). Please note that Supporting Information files do not need this step.

**Response:**

Thank you for your valuable advice. We used PACE to correct our pictures, which really helped us a lot.
